# Variation in social systems within *Chaetodon* butterflyfishes, with special reference to pair bonding

**DOI:** 10.1371/journal.pone.0194465

**Published:** 2018-04-11

**Authors:** Jessica P. Nowicki, Lauren A. O’Connell, Peter F. Cowman, Stefan P. W. Walker, Darren J. Coker, Morgan S. Pratchett

**Affiliations:** 1 Australian Research Council Centre of Excellence for Coral Reef Studies, James Cook University, Townsville, Queensland, Australia; 2 Department of Biology, Stanford University, Stanford, California, United States of America; 3 Red Sea Research Center, Division of Biological and Environmental Science and Engineering, King Abdullah University of Science and Technology, Thuwal, Saudi Arabia; Oregon Health and Science University, UNITED STATES

## Abstract

For many animals, affiliative relationships such as pair bonds form the foundation of society and are highly adaptive. Animal systems amenable for comparatively studying pair bonding are important for identifying underlying biological mechanisms, but mostly exist in mammals. Better establishing fish systems will enable comparison of pair bonding mechanisms across taxonomically distant lineages that may reveal general underlying mechanistic principles. We examined the utility of wild butterflyfishes (f: Chaetodontidae; g: *Chaetodon*) for comparatively studying pair bonding. Using stochastic character mapping, we provide the first analysis of the evolutionary history of butterflyfish sociality, revealing that pairing is ancestral, with at least seven independent transitions to gregarious grouping and solitary behavior since the late Miocene. We then formally verified social systems in six sympatric and wide-spread species representing a clade with one ancestrally reconstructed transition from paired to solitary grouping at Lizard Island, Australia. *In situ* observations of the size, selective affiliation and aggression, fidelity, and sex composition of social groups confirmed that *Chaetodon baronessa*, *C*. *lunulatus*, and *C*. *vagabundus* are predominantly pair bonding, whereas *C*. *rainfordi*, *C*. *plebeius*, and *C*. *trifascialis* are predominantly solitary. Even in the predominantly pair bonding species, *C*. *lunulatus*, a proportion of adults (15%) are solitary. Importantly, inter- and intra-specific differences in social systems do not co-vary with other previously established attributes, including parental care. Hence, the proposed butterflyfish populations are promising for inter- and intra-species comparative analyses of pair bonding and its mechanistic underpinnings. Avenues for further developing the system are proposed, including determining whether the aforementioned utility of these species applies across their geographic disruptions.

## Introduction

Social bonds are foundational to many animal societies [1, 2]. Arguably, the most extreme form of social bond is the pair bond—a selective, relatively pro-social and enduring affiliation between two individuals that is maintained beyond (or outside of) reproduction. Pair bonding has independently evolved numerous times within and across major vertebrate lineages [3–7], where it represents a defining feature of species-typical social structure [8], shapes aspects of parental [9–11] and mating [12, 13] strategies, and confers considerable adaptive benefits [3, 4, 11, 14, 15]. Research is increasingly focused on the mechanistic (e.g., neuroendocrine) basis of pair bonding, largely due to its implications for the biological mechanisms of human pro-sociality, anti-social psychological disorders [16, 17], and physical health [18]. However, most of what is known about the mechanistic basis of pair bonding comes from extensive studies on a single genus, *Microtus* voles (reviewed in [19–26]). A scarcity of complementary research among other organisms has led to little being known about the evolution pair bonding mechanisms across vertebrates, making it difficult to identify general principles for the sub-phylum. Moreover, in many current mammal and avian systems for comparatively studying pair bonding, variation in pair bonding is confounded with variation in other life-history attributes, making it difficult to identify causal mechanisms specifically for pair bonding. This problem is perhaps most severe for male mammalian model species, wherein pair bonding species are parental and non-pair bonding species are non-parental, resulting in little being known about the mechanisms of pair bonding independently from parental care in this sex. Expanding pair bonding systems to better include teleost fishes is a promising solution to these limitations, owing to their distant taxonomic relation to mammalian and avian systems [27], unparalleled species diversity [28], and extreme diversity in social systems, ecology, and behaviour [29, 30].

*In situ* behavioral observations on wild organisms are a critical first step towards establishing the existence and variation of social systems within and among species [31–34]. Species that exhibit inter-individual variation in social systems are particularly useful for comparatively identifying mechanisms of social system plasticity [35–37] when potential confounds such as geographic occurrence, life history, and behavioral ecology are controlled. Whereas, inter-species comparisons within a taxon can inform mechanisms underpinning social system evolution when controlling for the aforementioned confounds and phylogenetic relatedness [38–40]. These inter-species comparison also potentially illuminate principles for the taxon that may not be apparent in a single species [41, 42]. While systems for comparatively studying the mechanisms of pair bonding were originally limited to a single genus of mammal, *Microtus* voles [43, 44], additional comparative systems for other taxon within mammals and other major lineages have recently emerged: mammals: *Peromyscus* mice [45]; birds: *Coturnix* quails [46]; teleosts: *Neolamprologus*, *Telmatochromis* [47] and *Herichthys* [48] cichlids; and Hawaiian butterflyfishes [49]. If we are to understand the deep evolutionary history of pair bonding mechanisms and identify general principals, then additional model systems need to be established across major vertebrate lineages.

Teleost fishes offer many opportunities for comparatively studying social systems [30, 50]. Among vertebrates, the lineage is the most taxonomically diverse (~29,000 described species) [28] and displays extreme variation of social behavior among individuals and species [29, 30]. The family Chaetodontidae (butterflyfishes and bannerfishes; “chaetodontids”) is attractive for comparative research into pair bonding specifically. Chaetodontidae are widely distributed throughout the world’s oceans, occurring in all coral reef regions [51, 52]. The family includes at least 127 extant species [51], 77 of which predominantly occur in paired social groups (data sourced from [52–59], S1 Table), ostensibly accounting for ~21% of all reported pair bonding marine fishes (data sourced from [5, 7]). Their evolutionary history is also relatively well understood, with about 75% of the family represented in a dated molecular phylogeny [60, 61]. *Chaetodon* butterflyfishes have undergone rapid species diversification relatively recently (~ 16 million years ago) [61], resulting in 93 nominal extant species, among which the majority (59 spp.) predominantly occur in paired social groups (data sourced from [52–59]). Available data on select pairing species suggests that pairs exhibit partner fidelity. Partners have been shown to remain together for the full duration of monitoring studies, which range from several months to seven years [53, 62–66]. Such duration of partner fidelity can be considered prolonged to long-term, since *Chaetodon* spp. consistently live for more than 10 years [67]. By contrast, a minority of *Chaetodon* spp. predominantly occur in solitude or gregarious (three+ individual) groups [53, 68, 69], suggestive of species diversity in social systems. As species diversity in Chaetodontid behavioural ecology [55, 70–72], biogeography [73, 74], and species relatedness [61, 75, 76] is well established, comparisons of social systems can be made in a highly controlled manner. Importantly, all chaetodontids are broadcast spawners that effectively display no parental care [53, 77], and would therefore provide the first insights into the mechanisms of pair bonding that are independent from parental care.

Although there are numerous studies on Chaetodontidae social behaviour, surprisingly few studies have established species’ typical social systems [55], defined by a whole of interactions and relationships between individuals, such as social grouping, aggression, social bonding, and group sex composition [78]. Consequently, few comparative systems for studying pair bonding have been developed for the clade [49]. Additionally, comparative Chaetodontidae pair bonding systems are yet to be developed within a framework that considers the evolutionary history of sociality, since this remains unexamined within the group.

In the current study, we re-traced the evolutionary history of chaetodontid sociality using ancestral reconstruction analysis. We then sought to confirm inter- and intra-species variation in social systems (i.e., pair bonding vs. solitary living) among six species through *in situ* studies of wild populations. To do so, we focused on features that are routinely recognized as characteristic of pair bonding across taxa, that are useful for distinguishing pair bonding from non-pair bonding social systems, and that are ecologically relevant to butterflyfishes. These features include i) predominant group size of two individuals [69, 79–82], ii) selective affiliation with a distinct partner [21, 79, 80], which in the case of fishes may be expressed as proximate and parallel (i.e., “pair”) swimming [53, 83], iii) selective aggression towards non-partners [23, 34, 46, 62], iv) predominantly heterosexual pair composition [14, 31, 53, 83–85], and v) long-term partner fidelity/endurance [14, 53, 65, 66, 79–81, 86, 87].

*Chaetodon lunulatus*, *C*. *baronessa*, *C*. *plebeius*, *C*. *rainfordi*, *C*. *trifascialis* (Clade 3 (CH3); ingroup), and *C*. *vagabundus* (Clade 4 (CH4); outgroup) [61] were selected for examining inter- and intra-species variation in pair bonding, for several reasons. Firstly, available evidence suggests that these species might exhibit dichotomous social systems, with *C*. *baronessa*, *C*. *lunulatus*, and *C*. *vagabundus* possibly being predominantly pair bonding, and *C*. *rainfordi*, *C*. *plebeius*, and *C*. *trifascialis* possibly being primarily solitary [31, 55, 68, 69, 88]. Apart from *C*. *plebeius*, this apparent species diversity in social systems appears to be highly consistent throughout their geographic distributions (data sourced from [31, 53, 55, 68, 69, 85]). However, as with most chaetodontids, the social systems of these species at a given geographic location has largely been inferred from few social proxies (mostly predominant group size) (e.g., [55, 69, 85, 88]) rather than verified by quantitatively and holistically assessing a repertoire of social behaviors that cumulatively define social systems (*C*. *lunulatus* (= *trifasciatus*) at Yaeyama Islands notwithstanding [62, 63, 69, 89]). Hence, reliable assessments of social systems for most of these species remain absent. Secondly, these species are closely related congeners [61, 76] that are widely distributed throughout the Indo-/Western-Pacific region [28], wherein they can be found in relative abundance and co-occurring in sympatry [31, 69, 90]. In this study, we tested the prediction that three species (*C*. *lunulatus*, *C*. *baronessa*, and *C*. *vagabundus*) would predominantly occur in enduring heterosexual pairs that exhibit selective affiliation towards partners over non-partners, and selective agonism towards non-partners over partners. Conversely, we predicted that three species (*C*. *trifascialis*, *C*. *plebeius*, and *C*. *rainfordi*) would predominantly occur in solitude, and exhibit infrequent and indiscriminate affiliation with another individual. Finally, we predicted that for one species, *C*. *lunulatus*, individuals would occur in either enduring heterosexual pairs that exhibit selective partner affiliation and selective non-partner agonism; or in solitude, exhibiting infrequent and indiscriminate affiliation with another individual. Confirming the variation and evolutionary history of social systems (pair bonding vs. solitary living) within these populations would establish them as useful systems for comparatively studying pair bonding on both an inter- and intra-species level, and within an evolutionarily-informed manner.

## Materials and methods

### Evolutionary history of Chaetodontidae sociality

To conduct ancestral reconstruction of social group sizes in the family Chaetodontidae, the most completely sampled and dated phylogeny ([61]) was chosen for use. This phylogeny includes 95 of the 127 described species and is based on four mitochondrial and four nuclear genes. Briefly, the eight-gene dataset underwent Maximum Likelihood (ML) analysis in the program Garli [91], with the best ML topology chosen as a starting tree for Bayesian age estimation analyses with fossil calibrations in the program BEAST [92]. This resulted in a posterior distribution of dated trees, which were then summarized as a maximum clade credibility tree (MCC).

A literature search was conducted to classify the predominant social group size of all chaetodontid species as either ‘pairing’, ‘gregarious’ (forming groups of three or more) or ‘solitary’. From this literature search, 79 species were classified as pairing, 17 were classified as gregarious and 14 were classified as solitary. Of the remaining species, one species has been recorded as both pairing and gregarious (*Chaetodon gardineri* [52], although this species was not sampled in the phylogeny) and the sociality of 17 species remains unknown, so they could not be determined here. Overall, there were 20 species with group size data that were missing from the phylogeny. Species sampled in the phylogeny where no accurate determination could be made on group size (*Amphichaetodon melba*, *Chaetodon blackburnii*, *Prognathodes marcellae*, *P*. *aya*) were coded as having an equal probability of being in any of the three states, allowing their probable state to be reconstructed during the ancestral reconstruction analyses.

The evolutionary history of Chaetodontidae social grouping was explored using a stochastic character mapping [93] function from the R package phytools [94]. The stochastic character mapping procedure samples simulated histories of a trait across the evolutionary history of a phylogeny and can incorporate topological uncertainty by conducting the analyses across a distribution of trees. Using this method, we examined transition rates among sociality character states and highlight the temporal origins of group sizes. To begin, we ran 1000 stochastic character maps on the MCC tree of Cowman and Bellwood [61] using the ‘make.simmap’ function of phytools with Q = “mcmc” to sample the transition matrix (Q) from its posterior probability distribution. The mean transition matrix from this analysis was then used to infer the stochastic character mapping of 1000 tree topologies sampled from the posterior distribution of trees reconstructed in the Cowman and Bellwood [61] study. For each tree, 10 stochastic maps were generated resulting in 10,000 mappings. From this set of stochastic character maps, the average number of transitions among character states were calculated, and character histories were summarized as state probabilities on the internal nodes of the MCC tree.

### Study populations and site

For the six focal species, the co-occurring populations at Lizard Island, located in the northern section of Australia’s Great Barrier Reef (14^o^40’S, 145^o^27’E), were chosen for this study, because their feeding ecology [90, 95–97], territoriality [98], demography [67], and habitat associations [95] have been previously established and do not co-vary with predicted social systems. All field studies were conducted on the north-western side of the island, where there are numerous distinct platform reefs that are easily accessible. Only individuals that were at least 80% of average species-specific asymptotic body length and therefore likely reproductively mature [85] were considered. Studies were conducted at haphazard times between 0800–1800 hrs from January–May 2013–2015. All collections for this study followed Great Barrier Reef Marine Park Authority permit approvals: G10/33239.1, G13/35909.1, G14/37213.1; James Cook University General Fisheries permit 170251. Animal handling and sacrifice procedures for the study were designed to minimize animal suffering and were approved by James Cook University Animal Ethics committee (approval: A1874).

### Verifying inter- and intra-specific variation in social systems

#### Species-predominant group size

Social systems were first assessed by determining species’ predominant group sizes. For each species, group size frequencies were measured at five haphazardly selected reefs using six replicate 50 m X 4 m belt transects per reef. During surveys, each individual (or group of individuals) within the transect area was followed for a 5 min observation period. Group size was determined by the number of individuals (either one, two, or three+ individuals) that displayed proximate swimming (within 1.5 m distance) for at least 3 consecutive min during the 5 min observation period. Swimming distance was visually estimated after practicing accuracy on dummy fishes prior to the study. Sample sizes of observations varied in accordance with variation in abundance: *C*. *rainfordi* (n = 48), *C*. *plebeius* (n = 61), *C*. *baronessa* (n = 76), *C*. *lunulatus* (n = 98), *C*. *trifascialis* (n = 43), *C*. *vagabundus* (n = 55). To determine the predominant group size for each species, the total number of observations of different group sizes were pooled across reef sites and compared to a pre-defined uniform distribution that would be expected if individuals had no preference for any group size (33.33% of observations in each group size) using a chi square goodness-of-fit.

#### Within- and between-group agonism and affiliation

**Fig 1:**
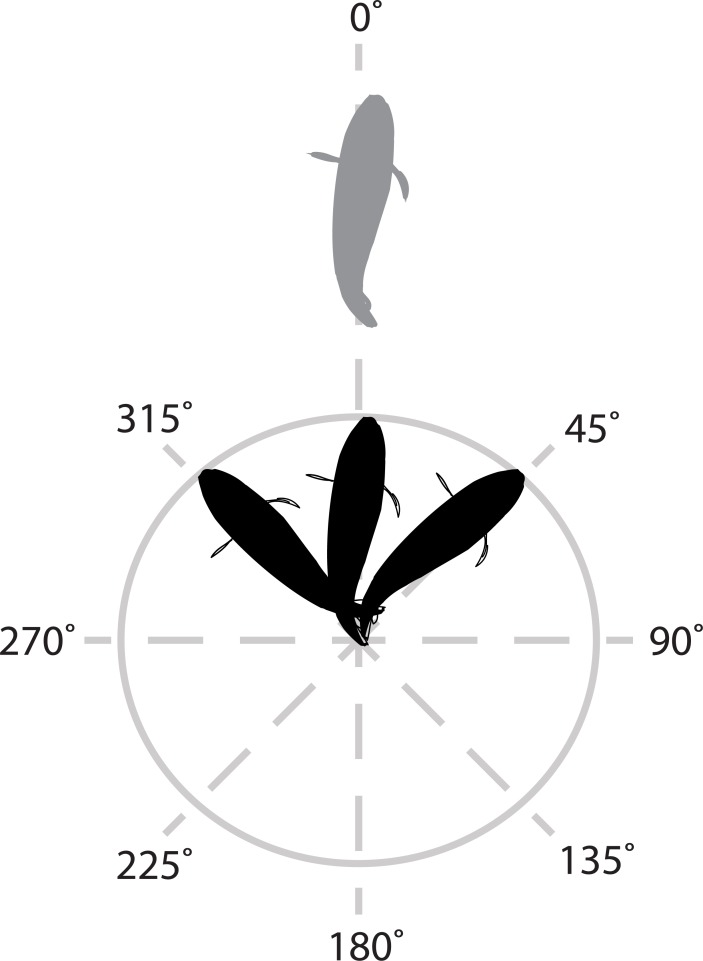
Schematic of parallel swimming examined in six *Chaetodon* species. Parallel swimming by the focal fish (black) was defined as being faced within a 315–45° angle relative to the faced position of the conspecific (grey), whose faced position was designated 0°.

To further explore social systems, field observations were conducted to measure social affiliation and agonism within and between conspecific groups. *In situ* behavioural observations were conducted on snorkel across five haphazardly selected reefs. Focal individual(s) within the group were identified and observed from a distance of 2–3 m. Focal individuals were allowed 3 min to acclimate to observers' presence. Time spent proximate swimming (defined as swimming within a 1.5 m distance from another conspecific) and parallel swimming (defined as swimming faced within a 315–45° angle relative to the faced position of another conspecific, whose faced position was designated 0° (Fig 1)), were sampled once every 10 sec throughout a 3 min observation. Swimming distance and angle were estimated visually. These behaviours were measured towards both partner and non-partner conspecifics for predominantly paired species, and towards other conspecifics for predominantly solitary species and solitary *C*. *lunulatus*. While we attempted to sample both proximate and parallel swimming for each fish observed, there were few cases in which only one of these behaviours were measured. Sample sizes of observations for each of proximate and parallel swimming behaviours are as follows: *C*. *rainfordi* (n = 14, each behaviour), *C*. *plebeius* (n = 15, each behaviour), *C*. *baronessa* (n = 18 and n = 20, respectively), paired *C*. *lunulatus* (n = 18, each behaviour), solitary *C*. *lunulatus* (n = 16, each behaviour), *C*. *trifascialis* (n = 15, each behaviour), and *C*. *vagabundus* (n = 24 and 17, respectively). For predominantly paired species, total agonistic acts, including staring, chasing, fleeing, and encircling (see [62] for detailed description) towards partners and non-partner conspecifics, were measured. Sample sizes for observations of agonistic acts were as follows: *C*. *baronessa* (n = 26), *C*. *lunulatus* (n = 25), *C*. *vagabundus* (n = 24). As it was determined in the present study that *C*. *lunulatus* is both pairing and solitary, for this species, proximate and parallel swimming with another conspecific was compared between paired and solitary individuals using non-parametric Mann-Whitney *U*-test (SPSS Software), due a lack of normality of residual variance.

#### Sex composition

To examine the sex composition of pairs among predominantly pairing species, a sub-sample of pairs were collected following behavioural observations and sacrificed. Individuals of predominantly solitary species and solitary *C*. *lulnulatus* were also collected for sex composition analysis. Gonads were removed and fixed in formaldehyde-acetic acid-calcium chloride solution (FACC) for at least one week. Thereafter, gonads were dehydrated in a graded alcohol series, washed in xylene, embedded in paraplast, sectioned transversely (7 μM thick), and stained with hematoxylin and eosin. Sections were examined under a compound microscope (400 X magnification) for the presence of characteristic sex cells [99, 100]. Among pairs, three categories of sex composition were found: heterosexual pairs, homosexual pairs, and pairs in which at least one individual was ostensibly a hermaphrodite. To statistically test whether paired individuals had a preference for partnerships of a particular sex composition, the number of pairs in different pair sex composition categories was compared to a pre-defined uniform distribution that would be expected if individuals had no preference for a given pair sex composition (33.33% of all pairs in each category) using a chi-squared goodness-of-fit.

#### Partner fidelity in pairs

To test whether the three predominantly pairing species of this study exhibit partner fidelity, we uniquely tagged pairs of each species (*C*. *baronessa*, n = 12; *C*. *lunulatus*, n = 18; *vagabundus*, n = 17) and then re-surveyed tagged fishes after six-weeks to record changes in partner identity (i.e., pair permutation). To facilitate re-detection of tagged fishes, this study was conducted on a single distinct platform reef, separated from nearby reefs by an open expanse of sand, which was expected to minimise movement of fishes away from the vicinity in which they were originally tagged. Preliminary visual surveys of the platform reef confirmed that for each species, at least 20 pairs occurred, providing opportunity for transient partnerships. Paired fishes were identified as described above, and then caught using a barrier net. Paired individuals were tagged on opposite sides of the dorsal musculature with unique and matching colour coded external tags using a hand-held tagging applicator (Floy T-bar Anchor) [101]. Tagged individuals were re-assessed for partner fidelity after six weeks, as this duration would inform the extent of short-term partner fidelity. A team of three snorkelers used an "expanding circle" search approach to reidentify tagged butterflyfishes. Once tagged fishes were detected, 3 min observations at a distance of at least 2 m from fish were again conducted to test for partner affiliation (as above); and respective partners were then carefully examined within 1 m to determine identity (i.e., tagged and known/untagged and unknown). We had planned to assess partner fidelity over several years, but this was not possible as no tagged fishes were re-identified at the 11-month reassessment within the study site. We observed multiple fish within the study site with scars at the tagging location and thus we assume that tags were dislodged from fishes, precluding further study.

## Results

### Evolutionary history of Chaetodontidae sociality

**Fig 2:**
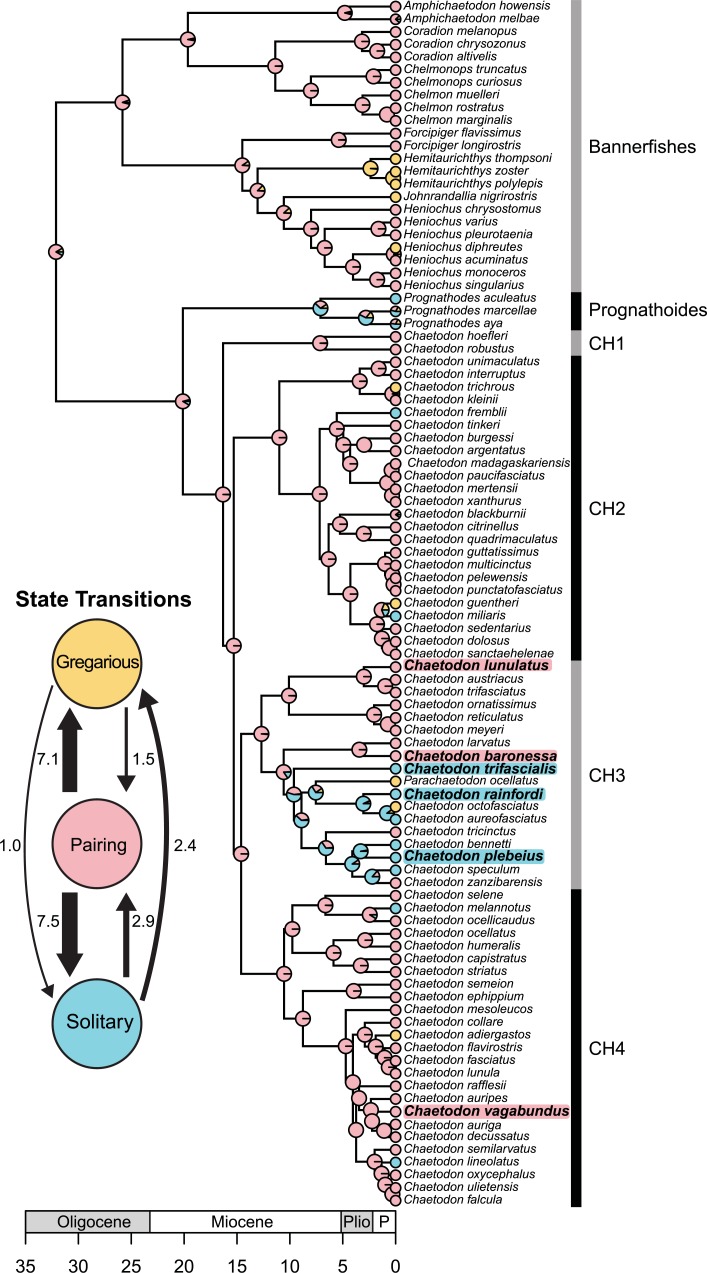
Ancestral reconstruction of social behavior in the family Chaetodontidae summarized on a published maximum clade credibility chronogram [61]. Pie charts at nodes represent the posterior probabilities of state reconstructions, summarized from 10,000 stochastic character maps across 1000 randomly sampled topologies from the BEAST posterior distribution of trees [61]. Within the family, pairing is reconstructed as the ancestral character with several subsequent independent transitions to solitary behavior or gregarious grouping and few reversals to pairing (inset). Within the study group (highlighted in blue for solitary and pink for pairing), pairing is ancestral, with one potential origin of solitary sociality in the common ancestor to *C*. *trifascialis*, *C rainfordi*, and *C*. *plebeius*. Time axis is in millions of years before present with major epochs identified (P: Pleistocene to Recent epoch).

Pair bonding is the most likely ancestral social system of the family based on the 10,000 stochastic character mappings summarized on an MCC tree (Fig 2). Several independent transitions were recorded from pair bonding to solitary (average of 7.5 transitions) and gregarious behaviour (average of 7.1 transitions) (Fig 2 inset). Reversions back to pair bonding from gregarious or solitary lineages appear to be uncommon. While a subclade within the *Chaetodon* Clade 3 (CH3) appears to retained the transition to solitary behaviour for much of its evolutionary history (with some changes to gregarious and pair forming), there was very little diversification observed within lineages reconstructed as displaying gregarious grouping. Gregarious behaviour is only reconstructed along extant, recent lineages across the greater *Chaetodon* clade (CH2, CH3, CH4), except for the expansion of the *Hemitaurichthys* lineage in the Bannerfish clade. Some species (two *Proganthodes* spp., *Amphichaetodon melbae* and *C*. *blackburnii*) were coded as having equal probabilities of being in either of the three states, due to lack of published observations. Based on their position in the phylogeny and the stochastic reconstruction, both *Proganthodes* species are reconstructed as mostly solitary, while both *Amphichaetodon melbae* and *C*. *blackburnii* are reconstructed as most likely pair bonding (Fig 2).

### Inter- and intra-specific variation in social systems

#### Species predominant group size

**Fig 3:**
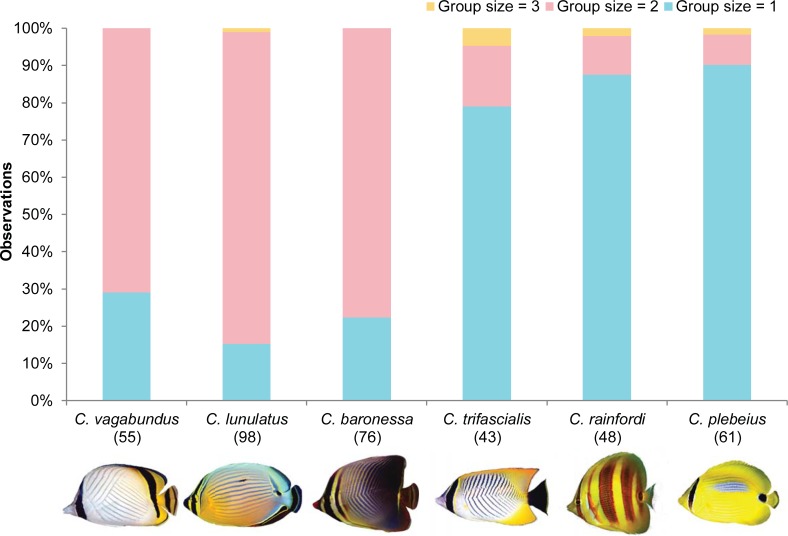
Group size frequency distribution of six *Chaetodon* spp. at Lizard Island. Numbers in parentheses indicate sample size of total observations of groups.

For all six species, the distribution of different group sizes differed significantly from uniform (*C*. *baronessa*: χ^2^ = 73, df = 2, *p* <0.001; *C*. *lunulatus*: χ^2^ = 114, df = 2, *p* < 0.001; *C*. *vagabundus*: χ^2^ = 42, df = 2, *p* < 0.001; *C*. *rainfordi*: χ^2^ = 64, df = 2, *p* < 0.001; *C*. *plebeius*: χ^2^ = 89, df = 2, *p* < 0.001; *C*. *trifascialis*: χ^2^ = 41, df = 2, *p* < 0.001). There was also an apparent dichotomy in predominant group size across species. Regardless of study site, *C*. *baronessa*, *C*. *lunulatus*, and *C*. *vagabundus* had a predominant group size of two individuals (78, 84, and 71% of individuals found in pairs, respectively) and were seldom found in a group size of one individual (22, 15, and 29% of observations, respectively) (Fig 3). Among predominantly pairing species, group sizes of three+ were only ever observed for *C*. *lunulatus* and only on one occasion. By contrast, *C*. *rainfordi*, *C*. *plebeius*, and *C*. *trifascialis* had a predominant group size of one individual (88, 90 and 80%, respectively) (Fig 3). Individuals of these species were less commonly observed paired (10–15%), and very rarely observed in a group size of three+ (1–2%).

#### Level of selective proximate and parallel swimming

**Fig 4:**
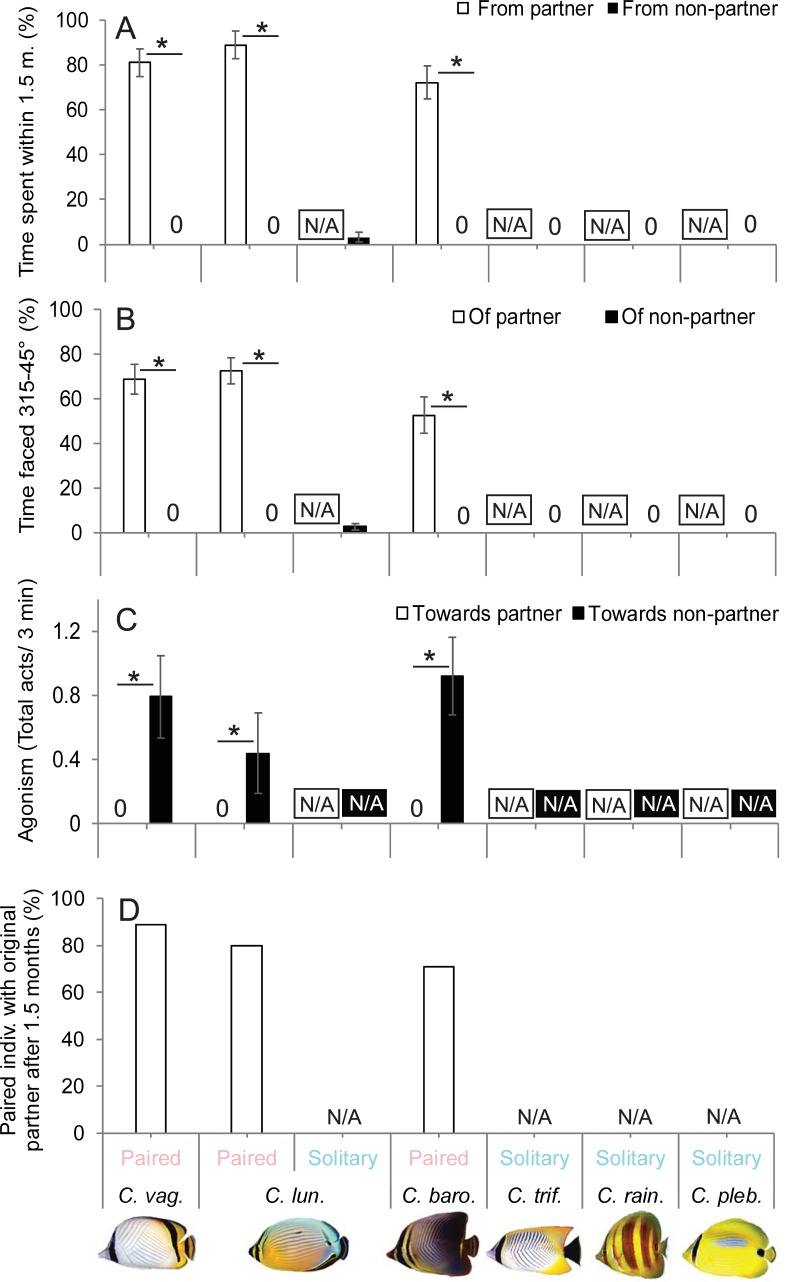
Differences in social behaviors between predominantly paired and solitary grouped *Chaetodon* spp. and *C*. *lunulatus* individuals. (A) Time spent proximate swimming with another conspecific (mean % ± standard error). (B) Time spent parallel swimming with another conspecific (mean % ± standard error). (C) Agonism towards partner vs. non-partner conspecifics among pairs (mean % ± standard error). (D) Percentage of pairs displaying partner fidelity after six weeks. Asterisks indicate significant differences between groups. N/A = not applicable.

The occurrence of proximate and parallel swimming clearly distinguished paired versus solitary grouped species (Fig 4A and 4B). Pairs of *C*. *baronessa*, *C*. *lunulatus* and *C*. *vagabundus* ranged as a single coordinated social unit throughout the reef, spending the majority of time swimming within 1.5 m of their partner (72 ± 7.41, 89 ± 6.2, and 81% ± 6.1 SE, respectively) and most of the time were faced within a 315–45° angle of their partner (53 ± 8.1, 72 ± 5.8 SE, and 69% ± 6.6 SE, respectively) (see Fig 1). By contrast, singletons of *C*. *rainfordi*, *C*. *plebeius*, and *C*. *trifascialis* displayed no apparent social affiliation with another individual, as they spent 100% of their time swimming further than 1.5 m from another conspecific; and most commonly, no other conspecific was within a field of view. Similarly, proximate and parallel swimming strongly varied between paired and solitary grouped *C*. *lunulatus* individuals (proximate swimming: *U* = 9, *p* < 0.001; parallel swimming: *U* = 9.5, *p* < 0.001) (Fig 4A and 4B**)**. While paired individuals displayed these behaviors exclusively with their partners at relatively high levels (swimming within 1.5 m from partner for 89% ± 6.2 SE of time; swimming faced within a 315–45° angle of their partner 72% ± 5.8 SE of the time), solitary individuals displayed these behaviors at relatively low levels (swimming within 1.5 m from another conspecific 3.1% ± 2.3 SE of time; swimming faced within a 315–45° angle of another conspecific 2.8% ± 1.5 SE of the time).

#### Level of selective agonism of pairs

Pairs of *C*. *baronessa*, *C*. *lunulatus*, and *C*. *vagabundus* displayed agonism exclusively towards non-partner conspecifics (Fig 4C), and no agonism towards partners. However, even agonism towards non-partner conspecifics was infrequent and minor, consisting mostly of staring displays.

#### Partner fidelity in pairs

Across the three pairing species, a total of 49 (52%) of the original 94 tagged fish were re-identified after six weeks: *C*. *baronessa*: 12/24 (50% of) fish, *C*. *lunulatus*: 19/36 (53% of) fish; *C*. *vagabundus*: 18/34 (53% of) fish, and within their original general reef location. *C*. *baronessa* was re-identified on seven occasions: five (71%) of which individuals were paired with their original partners, and two (29%) of which individuals were found solitary. *C*. *lunulatus* was re-identified on 10 occasions: eight (80%) of which individuals were paired with original partners, one (10%) of which individuals were paired with a different (non-tagged) fish, and one (10%) of which individuals were found solitary. *C*. *vagabundus* was re-identified on nine occasions: eight (89% of) of which individuals were paired with original partners, and one (11%) of which an individual was paired with a different (non-tagged) fish (Fig 4D). In cases where re-identified tagged fish were not found with their original partners, their original partners were not found by the observers.

#### Sex composition

Among predominantly pairing species, most of the pairs for which we determined sex histologically were heterosexual, whereas homosexual pairs, or pairs comprised of at least one ostensive hermaphrodite were uncommon (Table 1). The frequency of heterosexual pairs differed significantly from a uniform distribution (*C*. *baronessa*: *X*^2^ = 17.7, df = 2, *p* < 0.001*; C*. *lunulatus*: *X*^2^ = 19.2, df = 2, *p* < 0.001; *C*. *vagabundus*: *X*^2^ = 12.0, df = 2, *p* < 0.001). Among predominantly solitary species, singletons were mostly female and uncommonly male or ostensive hermaphrodites (Table 2). Among solitary *C*. *lunulatus*, singletons were equally male and female (Table 2).

**Table 1:** Predominantly pairing species in current study: Sex composition of pairs.

Species	Hetero. pairs	Homo. pairs	Hermaph.^ɫ^ pairs	Total pairs	Hetero. pair ratio	Homo. pair ratio	Hermaph. pair ratio
*C*. *baronessa*	12	2 (F + F)	0	14	0.86*	0.14	0.00
*C*. *lunulatus*	13	1 (M + M)	1 (F + H)	15	0.87*	0.07	0.07
*C*. *vagabundus*	6	0	0	6	1.00*	0.00	0.00

*Key*: F = female; Hetero. = heterosexual; H = Hermaph. = ostensive hermaphrodite; Homo. = homosexual; M = male.

* = significantly higher than by chance alone.

^ɫ^Pairs for which at least one partner was an ostensive hermaphrodite.

**Table 2:** Predominantly solitary species and solitary *C*. *lunulatus* in current study: Sex composition of individuals.

Species	Males	Females	Hermaph.	Total indiv.	Male ratio	Female ratio	Hermaph. ratio
*C*. *rainfordi*	2	12	1	15	0.13	0.80	0.07
*C*. *plebeius*	0	14	1	15	0.00	0.93	0.07
*C*. *trifascialis*	2	12	1	15	0.13	0.80	0.07
*C*. *lunulatus*	6	5	0	11	0.55	0.45	0.00

*Key*: Hermaph. = ostensive hermaphrodite.

## Discussion

### Evolutionary history of Chaetodontidae sociality

This is the first study of the evolutionary history of Chaetodontidae sociality. Pairing appears ancestral and moderately conserved within the family (Fig 2), with transitions to solitary and gregarious grouping occurring only in the last 10–15 million years. Potential factors associated with the evolutionary transition from pairing to solitary or gregarious behavior might stem from the expansion of coral reef habitat in the Indo-Australian Archipelago (IAA) that promoted subsequent species diversification during this time (the mid-Miocene)[61]. Pioneering populations expanding their geographic ranges to colonize distant locations may have been of exceptionally low density; precluding their ability to pair. Conversely, given that these populations may have been particularly vulnerable to predation during re-colonization, schooling as a means of anti-predation may have been favored. Finally, expansion of coral reef habitat may have diversified dietary options for butterflyfishes, thereby diversifying dietary-mediated social systems (see discussion below). Only in one instance did the transition to solitary behavior appear to have any subsequent diversification within a *Chaetodon* subclade (CH3, Fig 2). This appears to have occurred in a common ancestor of three of our study species that reside within clade CH3 (*C*. *rainfordi*, *C*. *plebeius*, and *C*. *trifascialis*). (Although solitude is the most probable ancestral state of these three species, this should be interpreted cautiously, given that the ancestral state probability of solitude is ca. 55%.). The CH3 clade also includes potential transitions to gregarious behavior (*Parachaetodon ocellatus*, and C. *octofasciatus*) and two independent reversions to pair bonding behavior (*C*. *tricintus*, *C*. *zanzibarensis*).

It is likely that the inclusion of unsampled Chaetodon species in future phylogenies might alter the character reconstructions highlighted here. However, based on the current schematic of the family, only three species are missing from clade CH3 (*C*. *triangulum*, *C*. *melapterus*, and *C*. *andamanensis*), all of which were found to be pairing. *C*. *andamanensis* is the only likely species to be placed within the CH3 subclade representing a transition to solitary behavior (most probably as a sister species to *C*. *plebeius*) [52], potentially representing another reversion to pair bonding within the clade.

### Intra- and inter-specific variation in social systems of *Chaetodon* butterflyfishes

This is one of the few studies to formally characterize the diversity of social systems within and among several butterflyfish species, including those inhabiting the same geographic location (see also [31, 102]). Results support our initial hypothesis that at Lizard Island, *C*. *baronessa*, *C*. *lunulatus*, and *C*. *vagabundus* are predominantly pair bonding, while *C*. *rainfordi*, *C*. *plebeius*, and *C*. *trifascialis* are predominantly solitary. They moreover meet our expectation that in *C*. *lunulatus*, both pair bonding and solitary living occurs among individuals. This reaffirms that butterflyfishes exhibit considerable diversity in social systems—an assumption that has been largely based on sparse behavioral observations, and primarily predominant group size [55, 69, 88].

#### Intra-specific variation in *Chaetodon lunulatus*

We found that at Lizard Island, *C*. *lunulatus* occurs in pairs 90% of the time. Heterosexual pairing predominates, occurring significantly more often than expected by chance alone, and therefore appears to be favored. Consistently, Pratchett et al [85] found that *C*. *lunulatus* adult pairs are predominantly heterosexual at Lizard Island (92%), presumably in order to facilitate reproduction [85]. We found that pairs display a high level of proximate and parallel swimming that occurs exclusively between partners. Even when partners were not swimming “proximately” (≤ 1.5 m), they almost always remain within close range (≤ ~4 m) of each other. While agonism in paired individuals is infrequent, it occurs exclusively towards non-partner conspecifics. Finally, pairs appear to be enduring, as all but one remained together for the full duration of the study (six weeks). While we had hoped to measure partner fidelity for a much longer time (> 12 months), this was not feasible, due to loss of tags after six weeks. In future studies, we suggest longer-term assessment of partner fidelity using unique, naturally-occurring markings on focal individuals for identification [63, 86] in preference to man-made tags. When taken together, these observations verify that *C*. *lunulaus* is predominantly and strongly pair bonding at Lizard Island.

Consistent with Lizard Island, other populations of *C*. *lunulatus* display a predominant social group size of two individuals. *Chaetodon lunulatus* exhibits the highest prevalence of paired grouping among butterflyfishes overall [55], where 95% of observations at Yaeyama Island (Japan) [69], 81% at Moorea Island (French Polynesia) [68], 84% at Heron Island (Australia) [31], 76% at Marshall Islands (Australia) [31], and 68% at Palm Island (Australia) [85] are of paired groups. Reese (1975) reported a relatively low pairing ratio (53%) at Johnston Island (Hawaii); however, this was from a relatively low sample size (n = 17 total observations). Among pairs, partners display highly affiliative pair swimming, maintaining coordination and close proximity while roaming throughout the reef [31], and particularly in a shared long-term territory [86, 89], which likely functions as a form of territory defense that conspicuously advertise occupancy [53]. Within the Yaeyama Islands (Japan) population, for example, partners spend 89% of their time swimming within 2 m of each other, and only 11% of their time swimming at further distances [89]. Aggression between partners rarely occurs, and when it does may be consequent of failed partner recognition [89]. By contrast, aggression towards non-partners is well documented in the species [31, 62, 72, 88, 98, 103], including the Lizard Island population [72, 88, 98], where it is attributed to territory defense [31, 72, 98, 103] and mate-guarding [62]. However, territorial aggression in *C*. *lunulatus* pairs is generally passive [69, 103], consistent with the ‘dear enemy’ model of low-cost resource defense once territories have been established among neighbors [14, 104]. Partner fidelity in *C*. *lunulatus* has been previously examined only once, where individuals remained paired with the same partner for up to seven years (Heron Island, Australia) [64]. Finally, partnerships are predominantly (93% of pairs, Palm Islands, Australia) [85], if not exclusively (100% of pairs, Heron Island, Australia) [31] heterosexual. Furthermore, one study has shown that mating occurs exclusively within the pair (Yaeyama Islands, Japan) [63]. Hence, *C*. *lunulatus* is predominantly and strongly pairing throughout its geographic range, and findings from specific populations suggest that these partnerships are both socially and reproductively monogamous.

At Lizard Island, we recorded that 15% of *C*. *lunulatus* adults occur in a group size of one individual that rarely exhibits proximate or paralleled swimming with another conspecific; and hence, are solitary. It is possible that in certain cases, paired individuals were mistaken for singletons. However, this would have occurred infrequently, since among pairs, partners spend nearly all their time swimming within 4 m of each other; and yet in nearly all instances where individuals were recorded as solitary, another conspecific was not within field of view. Similarly, among the Yaeyama Islands (Japan), Moorea Island (French Polynesia), Heron Island, Marshall Island (Australia), and Johnston Island (Hawaii) populations; solitary individuals occur on average 7% of the time. Elsewhere (Johnston Island, Hawaii), the proportion of solitary individuals is as high as 47% [31]. In any population, a small proportion of mature individuals would be expected to be solitary due to partner scarcity or loss [105]. It is also possible that there are differences in the propensity to pair bond vs. remain single within and between populations, due to differences in selective pressures (e.g., food competition).

#### Inter-specific variation among *Chaetodon* species

We found that at Lizard Island, *C*. *baronessa* and *C*. *vagabundus* occur in pairs (78 and 71% of observations, respectively), infrequently in solitude (22% and 29% of observations, respectively), and never in gregarious groups. In these species, heterosexual pairing predominates, and occurs significantly more frequently than that expected by chance alone, indicating that it is favored. Paired individuals of *C*. *baronessa* and *C*. *vagabundus* frequently and exclusively affiliate with their partners, and agonism is exclusively directed towards non-partner conspecifics and is generally passive (i.e., dominated by visual or lateral displays and chasing is uncommon). Pairs exhibit strong partner fidelity, with the majority maintaining their original partners throughout the duration of the study (six weeks). In the few cases where individuals were not found with original partners, original partners could not be found within the focal reef, which might indicate forced partner separation due to mortality. Overall, we verify that (as in *C*. *lunulatus*), *C*. *baronessa* and *C*. *vagabundus* are predominantly and strongly pair bonding at Lizard Island. Consistently, the predominant group size of *C*. *baronessa* and *C*. *vagabundus* is invariably paired across study populations. For *C*. *baronessa*, 70% of observations at Heron Island (Australia) [31] and 55% at Yaeyama Island (Japan) [69] are of paired groups. Whereas for *C*. *vagabundus*, 75% of observation at Yaeyama Island (Japan) and Moorea Island (French Polynesia) and 65% at Heron Island (Australia) populations are of paired groups. In both species, pairs maintain long-term territories that they defend against other butterflyfishes (*C*. *baronessa*: Heron Isl., Australia [31, 86]; *C*. *vagabundus*: Sesoko Isl., Japan [106]). However, previous descriptions of intra-pair relations are anecdotal, qualitative, and limited to one population of *C*. *baronessa* (Heron Island, Australia). Here, partners reportedly graze far apart within their territory, and only momentarily swim close together upon return from extra-territory forays [31]. For both species, pair sex composition has been previously examined in one population (Heron Isl., Australia), where all pairs are heterosexual [31]. Based on these aforementioned attributes of select populations and despite further descriptions of partner relations or fidelity for *C*. *baronessa* or *C*. *vagabundus*, both species are nevertheless presumed in the literature to be pair bonding throughout their distributions [55]. While never explicitly tested or observed in *C*. *baronessa* or *C*. *vagabundus*, the prevalence of pair bonding does imply monogamous mating [5, 55]. However, spawning observations (as per [63]) are required for verification.

By contrast, we found that at Lizard Island, *C*. *trifascialis*, *C*. *rainfordi*, and *C*. *plebeius* all occur primarily as solitary individuals (80, 88, and 90% of observations, respectively), and rarely in pairs (8–16% of observation) or aggregations (2–5% of observations). Singletons exhibit no apparent social affiliation with another conspecific, as they exclusively swim alone and often clearly beyond the visual range of other conspecifics. Across its geographic range, *C*. *trifascialis* predominantly occurs as solitary individuals (Red Sea: 93% of observations [53]; Moorea, French Polynesia: 86% [68]; Heron Isl., Australia: 82% [31]; Yaeyama Isl., Japan: 100% [69]. During the reproductive season, solitary grouping remains the predominant group size, but not surprisingly, its prevalence can modestly decline (and pairing can increase) (Yaeyama Isl., Japan) [69]. Adults establish long-term territories [86, 87], wherein territories of males encompass those of females [87]. In Kawashima (Japan), males repeatedly visit females within their territories, but spend only a short time swimming together [87]. They moreover mate sequentially with inhabiting females, suggestive of haremic mating [87]. Singletons aggress against same sex conspecifics as a form of mate guarding [87] and against other butterflyfishes as territory defense [31, 98]. Social grouping of *C*. *rainfordi* has been previously examined only at Heron Isl. (Australia) [31]; where solitary individuals occur 98% of the time. *Chaetodon pebeius* is also predominantly solitary at Heron Isl. (occurring 93% of the time), although the species exhibits no predominant group size at Yaeyama Isl., (Japan) where it occurs equally as solitary and paired individuals (50% of observations, respectfully). Together, these studies suggest that population-typical social system for the species is variable. Solitary living in *C*. *rainfordi* and *C*. *plebeius* may be attributed to their more generalized diet [90], which conceivably reduces competition and consequently the need for cooperative territory defense. Although mating systems of *C*. *rainfordi* and *C*. *plebeius* are yet to be studied, they have been considered by some researchers to be monogamous [5]. However, the preponderance of solitary living and female-biased sex ratio found here suggests they are either polygynous or polygamous [55]. Clearly, more work is required to establish *C*. *rainfordi* and *C*. *plebeius* mating systems.

Ultimate (current and evolutionary) explanations for variation in social systems shown among species in this study and generally apparent among *Chaetodons* remain poorly known. However, the prevailing view is that diversity in social systems is consequent of differences in diet specialization and of physiological (metabolic) constraints [83]. In general, pair bonding species predominantly if not exclusively consume coral [77, 83]. Since coral tends to have a relatively poor caloric value [107] and is inefficiently assimilated [56, 83]; dietary energy assimilation per bite may limit individual fitness in coralivorous butterflyfishes [83]. Yet, because coral is temporally and spatially stable, it is also capable of being defended. Hence, pair bonding may currently occur due to the adaptive benefits of co-operative resource (coral) defense [77, 83]. In support, observational and experimental studies show that pair bonded individuals confer higher feeding rates and energetic reserves than solitary counterparts [53, 108]. By contrast, gregarious species tend to feed on plankton, which may favor schools in search of this temporally and spatially unpredictable food source [83, 109]. Furthermore, because plankton blooms commonly occur in the open water, where there is increased vulnerability to predation; planktivory may further promote schooling as a means of anti-predation [77]. Solitary living on the other hand, is associated with consumption of benthic motile invertebrates with predator avoidance mechanisms, and it has been argued that these prey favor solitude because predators hunt for them more efficiently alone [110]. Whether dietary specialization drove the evolution of social system diversity now needs to be formally addressed using phylogenic approaches. What is currently apparent is that dietary specialization was not a selective force in the evolution of pairing behavior, as pairing behavior was already present in the Oligocene (~30 MYA), predating the origins of coralivory (Miocene: 15.7–3 MYA) [76].

Diet specialization alone cannot fully explain social system diversity across all *Chaetodontids*, however. This is certainly true for species of the current study, where one pair bonding species (*C*. *vagabundus*) is not a specialized corallivore, and all solitary species (*C*. *plebeius*, *C*. *rainfordi*, and *C*. *trifascialis*) are specialized corallivores [90]. Clearly, multiple current and evolutionary causes likely contribute to diversity in species-typical social systems within *Chaetodons;* including dietary specialization, spawning site preferences, predation pressure, territoriality, and population density; this is a topic requiring further investigation [111]. An unexpected and notable finding in this study was that one pair of *C*. *lunulatus* consisted of a female and of an individual simultaneously possessing both ovarian and testicular cells within their gonads. Gonads containing both sex cell types were also observed in one individual for each of the solitary species (*C*. *rainfordi*, *C*. *plebeius*, and *C*. *trifascialis*). A similar finding was previously reported for chaetodontids, in a pairing and monogamous congener, *C*. *multicinctus*, who was histologically shown to occasionally exhibit spermatogenic tissue within ovaries [66]. These results tentatively suggest sequential hermaphroditism [112, 113] in these species, challenging the currently held view that chaetodontids are invariably gonochoric [114]. The additional observation of female-biased sex ratios in the three solitary species in this study further suggests protogynous hermaphroditism in in these species. These findings provide impetus for further substantiating sex change within these species and exploring its possible adaptive function(s) in relation to their social systems, (e.g. [115, 116]).

### Utility of study species for comparatively studying regulatory mechanisms of pair bonding: Informing evolutionary history and controlling confounds

**Fig 5:**
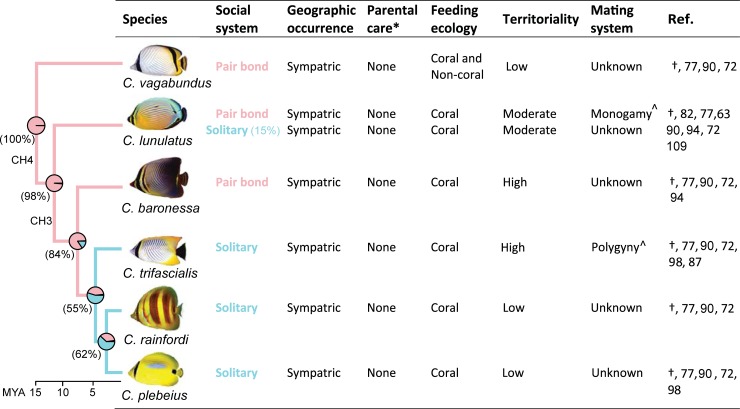
Dichotomous social systems (pair bonding vs. solitary living) among individuals of *Chaetodon lunulatus* and among species of *Chaetodon* at Lizard Island (current study) do not co-vary with other attributes (previously established), controlling for these variables while comparatively studying pair bonding. Phylogeny data sourced from [61], where species clades (CH) and ancestral state nodes and probabilities (in parentheses) are shown. *Notes*: † = current study. *Parental care is unstudied in Lizard Island populations and is presumed absent based on unequivocal reporting of pelagic spawning within Chaetodontidae. ^Mating systems of these populations at Lizard Island are presumed based on reports at other locations [63, 87].

Using stochastic character mapping and *in situ* behavioral observations, we have established that among the six study species, pair bonding is reconstructed as the ancestral state conserved in *C*. *lunulatus*, *C*. *baronessa*, and *C*. *vagabundus*, from which a single transition to solitary living occurred in the common ancestor of *C*. *rainfordi*, *C*. *plebeius*, and *C*. *trifascialis* (Fig 2). Such transitions from pair bonding to non-pair bonding systems are rare in animals and represent a unique opportunity to serve as a “natural knock-out” for comparatively identifying pair bonding mechanisms. While an earlier Chaetodontidae phylogeny [75] suggests that among these focal species, differences in social systems do not co-vary with relatedness, a more recent and more complete phylogeny [61] indicates phylogenetic non-independence. Species differences in social system, however, do not appear to co-vary with previously established species differences in biogeography or behavioral ecology (Fig 5). All species occur in sympatry at the study location, where they are benthic feeders that (with the exception of *C*. *vagabundus*) feed almost exclusively on scleractinian corals [90, 96] and exhibit differences in territoriality [72, 98] independently of differences in social system. Notably, these species, as in all butterflyfishes, are exclusively pelagic spawners, so are non-parental [110, 117, 118], offering a rare opportunity to examine pair bonding independently of this common confound [8, 119]. These controls also apply for pair bonded vs. solitary individuals of *C*. *lunulatus* (Fig 5). The control of parental care and territoriality while examining pair bonding is particularly important, because shared neuroendocrine mechanisms have been shown to regulate all three of these attributes [8]. Overall, the proposed design offers a unique opportunity for controlled intra- and inter-species comparative research on the regulatory mechanisms of pair bonding. A logical next step would be to sample wild fish and compare mechanistic components (e.g., brain regions, neural populations, and gene expression) between pair bonding and solitary *C*. *lunulatus* individuals and/or between pair bonding and solitary *Chaetodon* species, to identify mechanistic correlates of pair bonding within the clade.

Although the proposed *Chaetodon* butterflyfish system exhibits several attractive features for comparatively studying pair bonding, it does entail some limitations and challenges. As with most wild chaetodontids, most of these species have dietary requirements that are highly specialized and reliant on coral (*C*. *vagabundus* notwithstanding), making them difficult to maintain in captivity without growing coral. An alternative to growing coral could be to change the fishes’ diet to one that is more economical/accessible (e.g., crustaceans, mussels, *Aiptasia* spp.) which, although reportedly challenging, is achievable for even the most specialized corallivores, including *C*. *lunulatus* and *C*. *trifascialis* [120, 121]. While captive breeding of butterflyfish has been unsuccessful to date, it is expected to be achieved within the near future [120]. Until then, studies must be restricted to wild populations. Although these species are widely distributed and relatively common, we cannot be certain that our findings on their social systems at Lizard Island translate to all populations/geographic locations. However, available data on their predominant group size and social behavior is highly consistent across populations/geographic locations (*C*. *plebeius* notwithstanding), indicating their social systems are as well (*C*. *plebeius* notwithstanding once more). Verifying consistency in the social systems of these species across their geographic distributions should be a priority.

## Conclusions

In summary, this is the first study to examine the evolutionary history of Chaetodontidae sociality, revealing that within the family, pairing is ancestral and moderately conserved. It moreover verifies among six *Chaetodon* species at Lizard Island, Australia, a strong dichotomy in social systems representing one transition between them: from pair bonding in *C*. *lunulatus*, *C*. *baronessa*, *C*. *vagabundus* to solitary living in *C*. *trifascialis*, *C*. *rainfordi*, *C*. *plebeius*. These differences in social systems are not confounded with other life-history attributes, including parental care. Therefore, these populations are useful for conducting comparative analyses on the mechanistic correlates of pair bonding within a controlled and evolutionarily informed framework. A comparison of underlying biological mechanisms found within the group to those in other emerging/established teleost, avian, and mammalian systems (among whom pair bonding has evolved independently), will help illuminate both general and dissociable mechanisms of pair bonding within vertebrates.

## Supporting information

S1 Table Species-typical group sizes and mating systems in butterflyfishes. (DOCX)
