# Supplementary material for: Variation in social systems within Chaetodon butterflyfishes, with special reference to pair bonding
Source: PLoS One. 2018 Apr 11;13(4):e0194465. doi: 10.1371/journal.pone.0194465 (PMC5894994; doi:10.1371/journal.pone.0194465)
Supplement: S1 Table — (DOCX) [file pone.0194465.s001.docx]

| **S1 Table. Species-typical social group size and mating systems in Chaetodontidae.** | | | | |
| --- | --- | --- | --- | --- |
| **Genus** | **Species** | **Predominant Sociality (%)** | **Mating system** | **Reference** |
| *Amphichaetodon* | *howensis* | Pair |  | [1] |
| *Amphichaetodon* | *melbae* |  |  |  |
| *Chaetodon* | *adiergastos* | Gregarious |  | [1] |
| *Chaetodon* | *andamanensis* | Pair |  | [1, 2, 8] |
| *Chaetodon* | *argentatus* | Pair |  | [1, 2] |
| *Chaetodon* | *assarius* | Group |  | [1] |
| *Chaetodon* | *aureofasciatus* | Solitary (84.4%) |  | [4] |
| *Chaetodon* | *auriga* | Pair (73.3%) |  | [4] |
| *Chaetodon* | *auripes* | Pair (82.2%) |  | [4] |
| *Chaetodon* | *austriacus* | Pair (82.2%) |  | [4] |
| *Chaetodon* | *baronessa* | Pair (55.6%) |  | [4] |
| *Chaetodon* | *bennetti* | Solitary (86.1%) |  | [4] |
| *Chaetodon* | *blackburnii* |  |  |  |
| *Chaetodon* | *burgessi* | Pair |  | [3] |
| *Chaetodon* | *capistratus* | Pair (75%) | Monogamous | [4] |
| *Chaetodon* | *citrinellus* | Pair (85.6%) |  | [4] |
| *Chaetodon* | *collare* | Pair |  | [1] |
| *Chaetodon* | *daedalma* | Gregarious |  | [1] |
| *Chaetodon* | *declivis* |  |  |  |
| *Chaetodon* | *decussatus* | Pair |  | [1] |
| *Chaetodon* | *dialeucos* | Pair |  | [1] |
| *Chaetodon* | *dolosus* | Pair |  | [5] |
| *Chaetodon* | *ephippium* | Pair (66.7%) |  | [4] |
| *Chaetodon* | *excelsa* |  |  |  |
| *Chaetodon* | *falcula* | Pair (92%) |  | M. Pratchett, Unpub Data - Chagos |
| *Chaetodon* | *fasciatus* | Pair (40%) |  | [4] |
| *Chaetodon* | *flavirostris* | Pair |  | [1] |
| *Chaetodon* | *flavocoronatus* |  |  |  |
| *Chaetodon* | *fremblii* | Solitary | Polygynous | [4] |
| *Chaetodon* | *gardineri* | Pair-Gregarious |  | [1] |
| *Chaetodon* | *guentheri* | Gregarious |  | [1] |
| *Chaetodon* | *guttatissimus* | Pair (92%) |  | M. Pratchett, Unpub Data - Chagos |
| *Chaetodon* | *guyotensis* | Pair |  | [1] |
| *Chaetodon* | *hoefleri* | Pair |  | [1] |
| *Chaetodon* | *humeralis* | Pair |  | [1, 11] |
| *Chaetodon* | *interruptus* | Pair (77%) |  | M. Pratchett, Unpub Data - Chagos |
| *Chaetodon* | *jayakari* |  |  |  |
| *Chaetodon* | *kleinii* | Pair (80%) |  | [4] |
| *Chaetodon* | *larvatus* | Pair (52%) |  | [1], D. Coker, Unpub Data – Red Sea |
| *Chaetodon* | *leucopleura* | Solitary |  | [1] |
| *Chaetodon* | *lineolatus* | Solitary (47%) |  | [4] |
| *Chaetodon* | *litus* | Gregarious |  | [4] |
| *Chaetodon* | *lunula* | Pair (39%) |  | [4] |
| *Chaetodon* | *lunulatus* | Pair (95%) | Monogamous | [4, 9] |
| *Chaetodon* | *madagaskariensis* | Pair (100%) |  | M. Pratchett, Unpub Data - Chagos |
| *Chaetodon* | *marleyi* |  |  |  |
| *Chaetodon* | *melannotus* | Solitary (86.7%) |  | [4] |
| *Chaetodon* | *melapterus* | Pair |  | [1] |
| *Chaetodon* | *mertensii* | Pair |  | [1] |
| *Chaetodon* | *mesoleucos* | Pair |  | [1] |
| *Chaetodon* | *meyeri* | Pair (74%) |  | M. Pratchett, Unpub Data - Chagos |
| *Chaetodon* | *miliaris* | Solitary |  | [4] |
| *Chaetodon* | *mitratus* |  |  |  |
| *Chaetodon* | *modestus* | Gregarious |  | [6] |
| *Chaetodon* | *multicinctus* | Pair (83.3%) | Monogamous | [4, 7] |
| *Chaetodon* | *nigropunctus* | Pair (80%) |  | M. Pratchett, Unpub Data - Oman |
| *Chaetodon* | *nippon* | Gregarious |  | [1] |
| *Chaetodon* | *ocellatus* | Pair |  | [6] |
| *Chaetodon* | *ocellicaudus* | Pair |  | [1] |
| *Chaetodon* | *octofasciatus* | Gregarious |  | [1] |
| *Chaetodon* | *ornatissimus* | Pair (58.9%) | Monogamous | [4]; Hourigan, pers. obs., |
| *Chaetodon* | *oxycephalus* | Pair |  | [1] |
| *Chaetodon* | *paucifasciatus* | Pair (70%) | Monogamous | [4] |
| *Chaetodon* | *pelewensis* | Pair (73.3%) |  | [4] |
| *Chaetodon* | *pictus* | Pair |  | [1] |
| *Chaetodon* | *plebius* | Solitary (82.2%) |  | [4] |
| *Chaetodon* | *punctatofasciatus* | Pair (73.3%) |  | [4] |
| *Chaetodon* | *quadrimaculatus* | Pair (80%) | Monogamous | [4, 7] |
| *Chaetodon* | *rafflesi* | Pair (84.4%) |  | [4] |
| *Chaetodon* | *rainfordi* | Solitary (76.7%) |  | [4] |
| *Chaetodon* | *reticulatus* | Pair (58.3%) |  | [4] |
| *Chaetodon* | *robustus* | Pair |  | [1] |
| *Chaetodon* | *sanctaehelenae* | Pair |  | [6] |
| *Chaetodon* | *sedentarius* | Pair |  | [1] |
| *Chaetodon* | *selene* | Pair |  | [1] |
| *Chaetodon* | *semeion* | Pair |  | [6] |
| *Chaetodon* | *semilarvatus* | Pair (80%) |  | [12] |
| *Chaetodon* | *smithi* | Gregarious |  | [1] |
| *Chaetodon* | *speculum* | Solitary (72.2%) |  | [4] |
| *Chaetodon* | *striatus* | Pair (31%) |  | [13] |
| *Chaetodon* | *tinkeri* | Pair |  | [1, 2] |
| *Chaetodon* | *triangulum* | Pair (70%) |  | [4] |
| *Chaetodon* | *trichrous* | Gregarious |  | [1] |
| *Chaetodon* | *tricinctus* | Gregarious (70%) |  | [10] |
| *Chaetodon* | *trifascialis* | Solitary (93.3%) | Polygynous (Haeremic) | [4] |
| *Chaetodon* | *trifasciatus* | Pair (96%) |  | M. Pratchett, Unpub Data - Chagos |
| *Chaetodon* | *ulietensis* | Pair (57.2%) |  | [4] |
| *Chaetodon* | *unimaculatus* | Pair (72.2%) |  | [4] |
| *Chaetodon* | *vagabundus* | Pair (74.4%) |  | [4] |
| *Chaetodon* | *wiebeli* | Solitary |  | [6] |
| *Chaetodon* | *xanthocephalus* | Solitary |  | [6] |
| *Chaetodon* | *xanthurus* | Pair |  | [1] |
| *Chaetodon* | *zanzibariensis* | Pair (58%) |  | M. Pratchett, Unpub Data - Chagos |
| *Chelmon* | *marginalis* | Pair |  | [1] |
| *Chelmon* | *muelleri* | Pair |  | [8] |
| *Chelmon* | *rostratus* | Pair |  | [1] |
| *Chelmonops* | *curiosus* | Pair |  | [1] |
| *Chelmonops* | *truncatus* | Pair |  | [1] |
| *Coradion* | *altivelis* | Pair |  | [1] |
| *Coradion* | *chrysozonus* | Pair |  | [1] |
| *Coradion* | *melanopus* | Pair |  | [1] |
| *Forcipiger* | *flavissimus* | Pair |  | [1] |
| *Forcipiger* | *longirostris* | Pair |  | [1] |
| *Hemitaurichthys* | *multispinosus* |  |  |  |
| *Hemitaurichthys* | *polylepis* | Gregarious |  | [1] |
| *Hemitaurichthys* | *thompsoni* | Gregarious |  | [5] |
| *Hemitaurichthys* | *zoster* | Gregarious |  | [1] |
| *Heniochus* | *acuminatus* | Pair |  | [1, 2, 8] |
| *Heniochus* | *chrysostomus* | Pair |  | [1] |
| *Heniochus* | *diphreutes* | Gregarious |  | [1] |
| *Heniochus* | *intermedius* | Pair (76%) |  | [12] |
| *Heniochus* | *monoceros* | Pair |  | [1] |
| *Heniochus* | *pleurotaenia* | Pair |  | [1, 2] |
| *Heniochus* | *singularis* | Pair |  | [1] |
| *Heniochus* | *varius* | Pair |  | [1] |
| *Johnrandallia* | *nigrirostris* | Gregarious |  | [1] |
| *Prognathodes* | *aculeatus* | Solitary (91.1%) | Polygynous | [4] |
| *Prognathodes* | *aya* |  |  |  |
| *Prognathodes* | *basabei* | Gregarious |  | [14] |
| *Prognathodes* | *brasiliensis* |  |  |  |
| *Prognathodes* | *dichrous* | Pair |  | [1] |
| *Prognathodes* | *falcifer* |  |  |  |
| *Prognathodes* | *guezei* |  |  |  |
| *Prognathodes* | *guyanensis* |  |  |  |
| *Prognathodes* | *guyotensis* |  |  |  |
| *Prognathodes* | *marcellae* |  |  |  |
| *Prognathodes* | *obliquus* |  |  |  |

**References**

1. Kuiter RH. Butterflyfishes, bannerfishes, and their relatives: a comprehensive guide to Chaetodontidae & Microcanthidae: Twayne Publishers; 2002.

2. Allen GR, Steene RC, Humann P, DeLoach N. Reef Fish identification: Tropical Pacific. Jacksonville, Florida: New World Publications; 2003.

3. Allen G. Butterﬂy and Angelﬁshes of the World: Wiley, NY; 1979.

4. Reese ES. A comparative field study of the social behavior and related ecology of reef fishes of the family Chaetodontidae. Zeitschrift für Tierpsychologie. 1975;37(1):37-61.

5. Lieske E, Myers R. Collins Pocket Guide. Coral reef fishes: Indo-pacific and Caribbean: Haper Collins Publishers; 1994.

6. Allen GR, Steene RC, Allen M. A guide to angelfishes & butterflyfishes: Odyssey Publishing/Tropical Reef Research; 1998.

7. Yabuta S. Social groupings in 18 species of butterflyfish and pair bond weakening during the nonreproductive season. Ichthyological Research. 2007;54(2):207-10. doi: 10.1007/s10228-006-0391-x. PubMed PMID: WOS:000247456800010.

8. Randall JE, Allen GR, Steene RC. Fishes of the great barrier reef and coral sea. University of Hawaii Press. 1997.

9. Yabuta S. Spawning migrations in the monogamous butterflyfish, *Chaetodon trifasciatus*. Ichthyological Research. 1997;44(2):177-82. doi: 10.1007/bf02678695. PubMed PMID: WOS:A1997XG98500008.

10. Yabuta S, Berumen ML. Social structures and spawning behavior of *Chaetodon* butterflyfishes. In: Pratchett MS, Berumen ML, Kapoor BG, editors. Biology of butterflyfishes: CRC Press; 2014.

11. Liedke A, Segal B, Nunes LT, Burigo A, Buck S, Aburtooropeza O, et al. Feeding ecology of Chaetodon humeralis (Gunter 1860) in the Sea of Cortez, Mexico. ANA MARIA RUBINI LIEDKE. 2013:72.

12. Fricke HW. Pair swimming and mutual partner guarding in monogamous butterflyfish (pisces, chaetodontidae) - a joint advertisement for territory. Ethology. 1986;73(4):307-33. PubMed PMID: WOS:A1986F383900004.

13. Bonaldo R, Krajewski J, Sazima I. Meals for two: foraging activity of the butterflyfish Chaetodon striatus (Perciformes) in southeast Brazil. Brazilian Journal of Biology. 2005;65(2):211-5.

14. Pyle RL, Kosaki RK. *Prognathodes basabei*, a new species of butterflyfish (Perciformes, Chaetodontidae) from the Hawaiian Archipelago. ZooKeys. 2016;(614):137.
